# Multiparametric Assessment of Changes in Renal Tissue after Kidney Transplantation with Quantitative MR Relaxometry and Diffusion-Tensor Imaging at 3 T

**DOI:** 10.3390/jcm9051551

**Published:** 2020-05-21

**Authors:** Lisa C. Adams, Keno K. Bressem, Sonja Scheibl, Max Nunninger, Andre Gentsch, Ute L. Fahlenkamp, Kai-Uwe Eckardt, Bernd Hamm, Marcus R. Makowski

**Affiliations:** 1 Department of Radiology, Charité, Charitéplatz 1, 10117 Berlin, Germany; Sonja.scheibl@charite.de (S.S.); Maximilian.nunninger@charite.de (M.N.); Ute.fahlenkamp@charite.de (U.L.F.); Bernd.hamm@charite.de (B.H.);; 2 Department of Radiology, Charité, Hindenburgdamm 30, 12203 Berlin, Germany; 3 Department of Nephrology, Charité, Charitéplatz 1, 10117 Berlin, Germany; Andre.gentsch@charite.de (A.G.); Kai-uwe.eckardt@charite.de (K.-U.E.); 4 Department of Diagnostic and Interventional Radiology, Technical University of Munich, School of Medicine, 81675 Munich, Germany

**Keywords:** transplantation, renal pathology, renal biopsy, multiparametric MRI, quantitative tissue analysis

## Abstract

Background: Magnetic resonance relaxometry (MRR) offers highly reproducible pixel-wise parametric maps of T1 and T2 relaxation times, reflecting specific tissue properties, while diffusion-tensor imaging (DTI) is a promising technique for the characterization of microstructural changes, depending on the directionality of molecular motion. Both MMR and DTI may be used for non-invasive assessment of parenchymal changes caused by kidney injury or graft dysfunction. Methods: We examined 46 patients with kidney transplantation and 16 healthy controls, using T1/T2 relaxometry and DTI at 3 T. Twenty-two early transplants and 24 late transplants were included. Seven of the patients had prior renal biopsy (all of them dysfunctional allografts; 6/7 with tubular atrophy and 7/7 with interstitial fibrosis). Results: Compared to healthy controls, T1 and T2 relaxation times in the renal parenchyma were increased after transplantation, with the highest T1/T2 values in early transplants (T1: 1700 ± 53 ms/T2: 83 ± 6 ms compared to T1: 1514 ± 29 ms/T2: 78 ± 4 ms in controls). Medullary and cortical ADC/FA values were decreased in early transplants and highest in controls, with medullary FA values showing the most pronounced difference. Cortical renal T1, mean medullary FA and corticomedullary differentiation (CMD) values correlated best with renal function as measured by *e*GFR (cortical T1: *r* = −0.63, *p* < 0.001; medullary FA: *r* = 0.67, *p* < 0.001; FA CMD: *r* = 0.62, *p* < 0.001). Mean medullary FA proved to be a significant predictor for tubular atrophy (*p* < 0.001), while cortical T1 appeared as a significant predictor of interstitial fibrosis (*p* = 0.003). Conclusion: Cortical T1, medullary FA, and FA CMD might serve as new imaging biomarkers of renal function and histopathologic microstructure.

## 1. Introduction

Kidney transplantation is currently considered the best available therapy for patients with end stage renal disease [1]. However, temporary restriction or interruption of blood flow and restoration of blood supply during the transplantation procedure provoke a cascade of potential adverse events, leading to inflammation, edema, and tubular epithelial dysfunction [2,3]. Ischemia–reperfusion injury (IRI) is the main reason for delayed graft function and is most common in transplants from deceased donors, which account for the majority of transplants [4]. IRI often results in acute kidney injury (AKI), which is characterized by an abrupt decrease in the glomerular filtration rate (GFR), causing retention of metabolic waste and fluid as well as changes in electrolyte and acid–base balance [5]. Severe AKI also increases the risk of chronic kidney disease [6].

At present, diagnosis of renal impairment is predominantly based on serum creatinine (sCr) and the level of urine output [7]. However, sCR as a biomarker is associated with limitations, as it does not change until approximately half of the kidney function is lost and is potentially insensitive to rapid changes in kidney function, whereby the time delay between renal injury and an increase in sCR might result in missing therapeutic options [8]. In addition, sCR depends on a number of non-renal factors, including age, sex, muscle mass, hydration status, medications, and protein intake. Therefore, new measurements are needed to support the diagnosis of AKI, as early detection of AKI could improve the individual treatment and prognosis of the patient. In this context, the American Society of Nephrology specifically suggested the development of biomarkers for early detection of AKI as one of the research priorities. Both tubular atrophy and interstitial fibrosis are potential causes of renal transplant dysfunction [9]. However, the current reference standard for diagnosis is percutaneous biopsy, involving the risk of post-procedural complications and sampling errors [10].

Recent years have witnessed a growing interest in quantitative magnetic resonance imaging (MRI) of the kidney [11]. Magnetic resonance relaxometry (MRR) was not only shown to be feasible, but to offer highly reproducible pixel-wise parametric maps of tissue-specific T1 and T2 relaxation times, which may be used for non-invasive assessment of parenchymal changes caused by kidney injury and graft dysfunction. Although magnetic resonance relaxometry (MRR) is already frequently used in other organs (e.g., heart MRI) for assessment of edema, or fibrosis, the use of renal MRI is still relatively rare [12,13,14].

Another promising functional imaging technique for renal transplants is diffusion-weighted imaging (DWI). DWI enables measurement of the Brownian motion of water in the extracellular space, whereby the quantitative mean apparent diffusion coefficient (ADC) may be calculated from diffusion-weighted images. Going beyond that, information from DTI also contains the directionality of molecular motion. This is important for kidney imaging because anatomical structures such as vessels and tubules show a radial orientation, causing anisotropy. Directionality of molecular motion may be quantified by fractional anisotropy (FA). Prior research suggests that DWI is particularly sensitive to changes in the renal interstitium, such as renal fibrosis, cellular infiltration or edema [15]. For renal fibrosis, previous studies have consistently shown a negative correlation between ADC values and the amount of fibrosis [16,17]. Apart from reduced perfusion and tubular flow, this results from the deposition of fibrotic matrix in the interstitium, decreasing the Brownian motion of water molecules by collision [15].

The present study aimed to evaluate both quantitative T1 and T2 and cortical and medullary FA values as functional MRI biomarkers in patients short and long term after kidney transplantation to investigate potential acute and chronic post-transplantation kidney damage, using a field strength of 3 Tesla. Furthermore, a correlation of renal T1, T2, and DTI parameters with *e*GFR was investigated.

## 2. Experimental Section

### 2.1. Study Design and Population

This prospective study was approved by the local Institutional Review Board, whereby written informed consent was obtained from all participants in advance (reference number EA1/028/19). Between January 2019 and January 2020, 62 subjects received an MRI examination of the abdomen at a field strength of 3 T (mean age 55 ± 13 years, 47 males, 15 females). The study population consisted of 22 early transplants (55.0 ± 13.4 years, 17 males, 5 females), 24 late transplants (at least 3 months since transplantation) (51.0 ± 12.9 years, 20 males, 4 females), and 16 controls with healthy kidney function (59.9 ± 14.4, 10 men, 6 women), defined by an individual eGFR above 60. All subjects agreed to the participate in the study and had no contraindications against MRI. One patient was excluded due to insufficient image quality with multiple breathing artifacts. In the group of early transplants, sCR was determined the day before and after surgery, the day of the MRI examination, and 3 months after surgery. eGRF was used to estimate kidney function, based on the CKD-EPI (Chronic Kidney Disease Epidemiology Collaboration) formula [18].

In 7 patients, a prior percutaneous biopsy within a year of the MRI had been performed (time interval between biopsy and MRI: 148 ± 25 days). For these patients, Banff scores (according to the Banff Classification of Renal Allograft Pathology) for tubular, vascular, and glomerular involvement were obtained from the histopathologic report and then analyzed. The Banff scores included the lesion scores ct for tubular atrophy, ci for interstitial fibrosis, g for glomerulitis, i for interstitial inflammation, and ti for total inflammation.

### 2.2. Imaging Protocol

All examinations were performed using a 3-T clinical scanner (Magnetom VIDA, Siemens Medical Solutions, Erlangen, Germany) with an 18-channel body coil combined with a 32-channel spine coil and an automatic selection of coil elements. The patients received an MRI of the abdomen with sequences for anatomical imaging, including a half Fourier singleshot turbo spin-echo (HASTE) sequence with and without fat saturation and a Dixon sequence. HASTE is a single-section T2-weighted sequence with image acquisition in less than 1 s, which allows for breathing independent acquisition of high-resolution T2-weighted images. It is based on a single-shot technique to acquire sufficient data for the image from a single time of repetition. The short acquisition time makes it less susceptible to motion, which is especially helpful for abdominal imaging [17]. The Dixon technique is an MRI sequence, which is based on chemical shift imaging, and enables the simultaneous acquisition of water-only and fat-only images [18]. It can therefore reduce total scan time compared to conventional MRI sequences. For multiparametric imaging, a native steady-state precession readout single-shot Modified Look-Locker inversion recovery (MOLLI) sequence was used for T1 relaxometry and a spoiled gradient sequence (GRE) with an initial T2 preparation module was used for T2 relaxometry. Furthermore, an isotropic DTI sequence with 64 diffusion directions was acquired.

The T1 relaxometry technique is based on the acquisition of single-shot TrueFISP images with different inversion times (TI) after an initial inversion pulse. The acquisitions are assigned to the same cardiac phase, which allows a pixel-based assessment of the T1 value in the kidney. Each slice for T1 and T2 mapping was acquired within one breath-hold. Refer to Table 1 for tabulated magnetic resonance imaging parameters.

T1 and T2 maps were calculated automatically on a pixel-by-pixel basis. The resulting pixel-by-pixel maps had customized color coding with bright colors corresponding to longer and dark colors to shorter relaxation times. T1 and T2 maps are presented along with a color bar, corresponding to relaxation times between 0 and 2000 ms for T1 mapping and relaxation times between 0 and 120 ms for T2 mapping. ADC and FA maps were also calculated inline, with calculation of FA parametric maps to assess the degree of diffusion anisotropy. FA values enable a quantitative measurement of diffusion anisotropy on a scale from 0 (isotropic) to 1 (fully anisotropic).

In early transplants, MRI was performed 3–14 days after kidney transplantation. For late transplants, MRI was performed in patients within 0.5–13 years since transplantation.

In a small subgroup of 6 freshly transplanted patients, two MRI measurements were performed, the first between the Postoperative Days 4 and 6 and the second between Postoperative Days 11 and 14.

### 2.3. Imaging Analysis

All images were analyzed using PACS workstations (Centricity Radiology; GE Healthcare, Milwaukee, USA). Two radiologists performed the image analysis, blinded to the clinical information. They randomly evaluated T1, T2, ADC, and FA maps in two different reading sessions. For each patient, six ROIs were drawn in three slices for each cortex and medulla, a total of 18 ROIs for the cortex and 18 ROIs for the medulla (cf. Figure S1). The ROIs for cortex and medulla had uniform sizes per region. For T1 and T2 maps, which were acquired in oblique coronal plane, ROIs were copied from the T1 maps into the corresponding slices in the T2 maps. Manual adjustments were made where regions between the T1 and T2 maps did not match. For FA and ADC maps, which were acquired on the axial plane, ROIs were copied from the FA maps into the corresponding slices in the ADC maps to ensure quantitative measurements in matching regions of the medulla/cortex. The mean ROI size was 45.1 ± 17.6 mm^2^, the maximum size was 88.3 mm^2^, and the minimum size was 32.1 mm^2^. The pixel range for the cortex and medullary measurements was 612–2412 per measurement of the renal cortex area (5–134 per ROI) and 612–1584 per measurement of the renal medulla (5–88 per ROI). Measurements were averaged over two observers and then mean and standard deviations were calculated. In patients with diminished corticomedullary differentiation (CMD), anatomical T1 and T2 images in coronal planes were used for anatomical reference. CMD was calculated for T1, T2, ADC and FA values.

### 2.4. Statistical Analysis

All variables are given as mean values ± standard deviations. Student *t*-tests were used to assess the differences in means for continuous variables. Bar plots with error bars representing one standard deviation were used to represent the distribution of the mean T1 and T2 values in the different groups. A linear regression analysis was used to assess relationships between two measurements. A ROC analysis was performed to establish the suitability of different relaxometry and DTI parameters for the assessment of high and severe renal insufficiency. A logistic regression analysis was performed to assess the association between biopsy results and functional MR parameters. A *p*-value < 0.05 was considered statistically significant. Correction for multiple testing was performed based on the Holm–Bonferroni method [19]. Statistical analysis was performed with the statistical software “R” (Version 3.6.2, R Core Team, 2019, Vienna University of Economics and Business, Vienna, Austria).

## 3. Results

A total of 46 patients and 16 controls met the eligibility criteria, with a mean age of 52.9 ± 11.9 years for the transplanted patients and of 59.9 ± 14.4 years for the controls. Table 2 presents an overview of the study characteristics.

The average time interval between surgery and MRI was 6.8 ± 3.4 days for the early transplants and 5.5 ± 5.1 years for the late transplants. Kidney size was not significantly different between allografts with normal and decreased renal function (*p* > 0.05).

### 3.1. T1 Relaxometry

**Figure 1:**
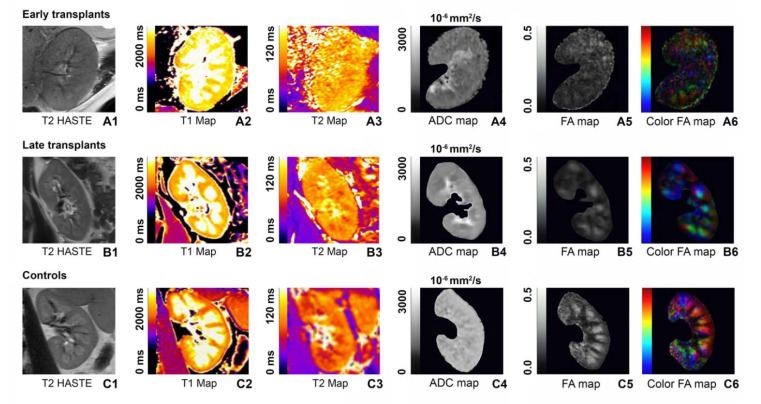
(**A1**–**A6**) Standard macroscopic T2-weighted sequence (**A1**) and images of renal T1 (**A2**) and T2 maps (**A3**) as well as ADC (**A4**) and FA maps (**A5**,**A6**) for an early transplant. (**B1**–**B6**) Images of a late transplant (half a year since transplantation). (**C1**–**C6**) MR images of a control with a healthy native kidney. Corresponding color bars are presented on the left side of each map, with 0–2000 ms for T1 maps, 0–120 ms for T2 maps, 0–3000 × 10^−6^ mm^2^/s for ADC maps, and 0.0–0.5 for FA maps. Generally, lighter colors/greyscales indicate higher values. As one can see from the above color-coded images, T1 and T2 relaxation times are highest in the newly transplanted kidney (**A1**–**A3**) and lowest in the healthy native kidney (**C1**–**C3**). In contrast, the ADC and especially the FA map for the newly transplanted kidney shows the lowest ADC and FA values with only moderate differentiation between cortex and medulla (**A4**,**A5**), while ADC and FA values are higher for the older stable allograft (**A4**,**B5**) and highest for the healthy native kidney (**C4**,**C5**).

The highest mean cortical T1 relaxation times were measured in early transplants with 1700 ± 53 ms, followed by late transplants with 1615 ± 47 ms and controls with 1514 ± 29 ms. All differences between the groups were significant for cortical T1, with early transplants showing higher T1 relaxation times compared to late transplants (*p* < 0.001) and controls (*p* < 0.001) (cf. Table 2 for an overview of the measured T1 values). Medullary T1 relaxation times were also highest in early transplants, followed by late transplants and controls. Again, the differences between early transplants and late transplants (*p* = 0.041) as well as the differences between early transplants and controls (*p* < 0.001) and between late transplants and controls (*p* = 0.003) were significant. As a result of the greater increase in cortical T1 relaxation times compared to medullary T1 relaxation times, the CMD was most lowered in early transplants (347.8 ± 119.3 ms), compared to late transplants (389.6 ± 117.5 ms) and controls (424.4 ± 86.8 ms, *p* = 0.046). However, these differences in T1 CMD were not significant. In addition, there were no significant differences depending on age or gender. Figure 1 shows an exemplary illustration of T1 and T2 maps in early transplants, late transplants and controls.

### 3.2. T2 Relaxometry

**Figure 2:**
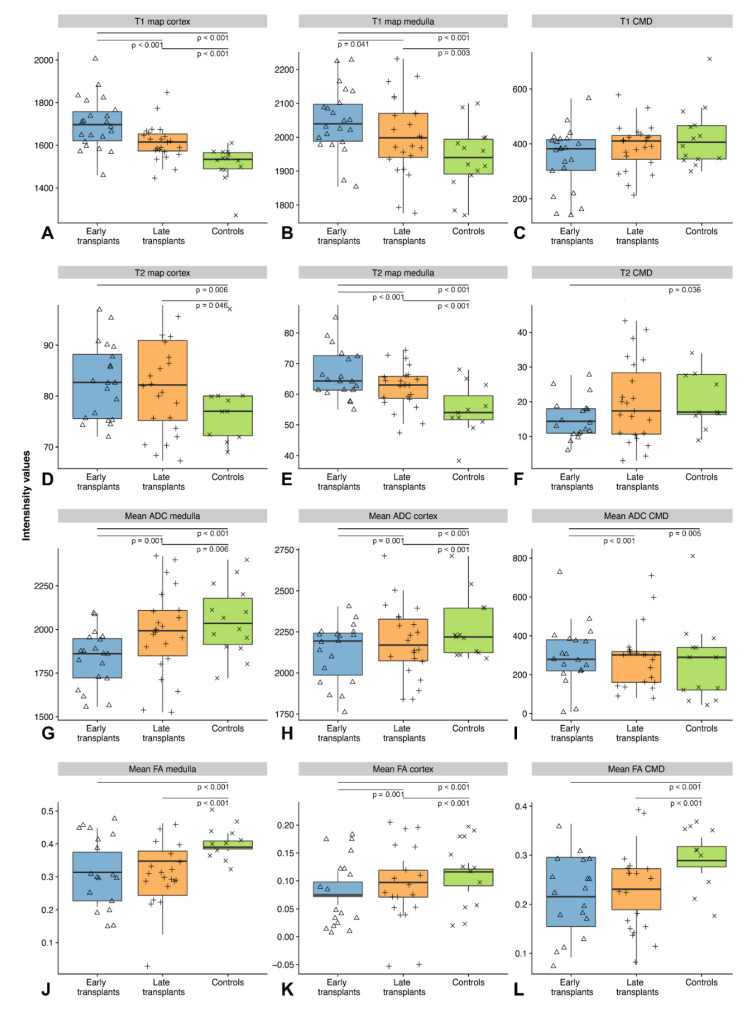
Overview of cortical and medullary T1 and T2 relaxation times, corticomedullary differentiation (CMD) and mean cortical and medullary ADC and FA values across the three groups (early transplants, late transplants, controls): (**A**) the distribution of cortical T1 relaxation times; (**B**) the distribution of medullary T1 relaxation times; (**C**) the distribution of T1 CMD values; (**D**) the distribution of cortical T2 relaxation times; (**E**) the distribution of medullary T2 relaxation times; (**F**) shows the distribution of T2 CMD values; (**G**) the distribution of mean medullary ADC values; (**H**) the distribution of mean cortical ADC values; (**I**) the distribution of ADC CMD values; (**J**) the distribution of mean medullary FA values; (**K**) the distribution of mean cortical FA values; and (**L**) the distribution of FA CMD values. Each graph displays the distribution of the respective parameter across early transplants, late transplants and controls with boxplots as well as the individual data points. Significant differences are indicated by the display of *p*-values.

Similar to T1 relaxometry, the highest cortical T2 relaxation times were measured in early transplants with 83 ± 6 ms, followed by late transplants with 82 ± 8 ms and controls with 78 ± 4 ms. Compared to T1 relaxometry, the differences between the groups were smaller between the groups and differences in medullary T2 relaxation times were more pronounced than differences in cortical T2 relaxation times. For T2 medullary relaxation times, the differences between early and late transplants (*p* < 0.001) and between early transplants and controls (*p* < 0.001) were significant. In addition, the difference between late transplants and controls reached significance level (*p* < 0.001). Correspondingly, the CMD was most lowered in early transplants (15.3 ± 7.8 ms), compared to late transplants (19.8 ± 8 ms, *p* = 0.06) and controls (22.8 ± 6.7 ms, *p* = 0.006). Only the T2 CMD between early transplants and controls reached significance (*p* = 0.036). Figure 2 shows an overview of the distribution of T1, T2, ADC, and FA values across early transplants, late transplants and controls.

### 3.3. Diffusion-Tensor Imaging

Mean ADC values from the renal cortex and medulla were highest in controls (ADC for the renal medulla: 2051 ± 49 10^−6^ mm^2^/s), followed by late transplants (ADC for the renal medulla: 1988 ± 63 10^−6^ mm^2^/s). Early transplants demonstrated the lowest ADC values (ADC for the renal medulla: 1834 ± 40 10^−6^ mm^2^/s) (cf. Table 2). Differences between all groups were significant. Similarly, cortical and medullary FA values were highest in controls (mean cortical FA: 0.093 ± 0.008; mean medullary FA: 0.401 ± 0.006), followed by late transplants (mean cortical FA: 0.108 ± 0.005; mean medullary FA: 0.318 ± 0.022), with the lowest values to be measured in early transplants (mean cortical FA: 0.085 ± 0.006; mean medullary FA: 0.310 ± 0.006). Differences between early transplants and controls and late transplants and controls were significant for mean medullary FA (both *p* < 0.001). For cortical FA, differences between all groups were significant (see Figure 2 for detailed overview of boxplots and corresponding *p*-values).

### 3.4. Association between T1 and T2 Relaxation Times and Cortical and Medullary FA Values and Renal Function

**Figure 3:**
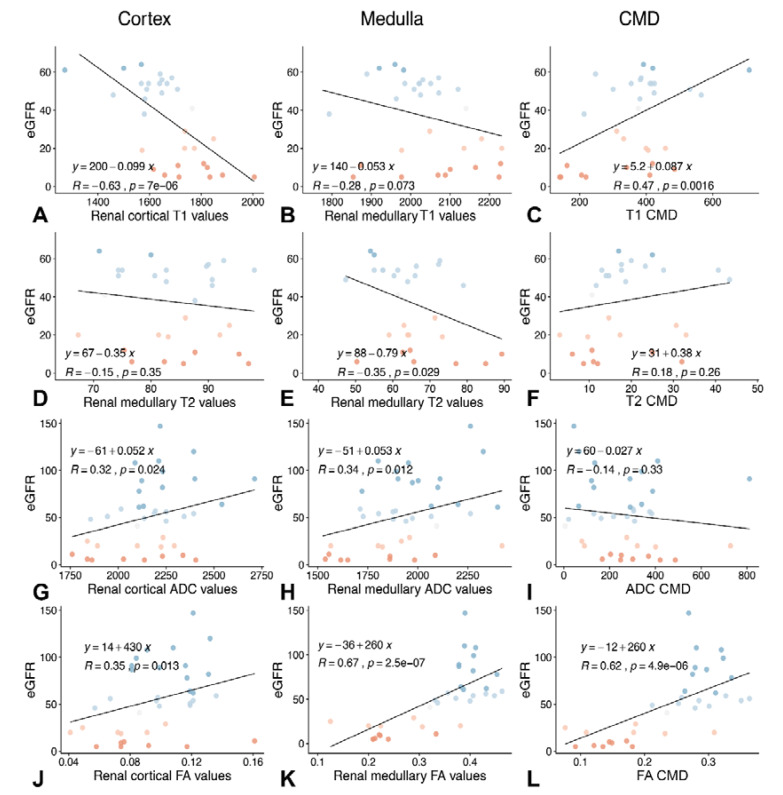
Correlation of cortical (left column), medullary (middle column) and corticomedullary differentiation parameters (right column) with *e*GFR. The first row (**A**–**C**) shows the correlation between T1 parameters (cortical T1 values (**A**); medullary T1 values (**B**); and T1 corticomedullary differentiation (CMD) (**C**)) and *e*GFR. The second row (**D**–**F**) shows the correlation between T2 parameters (cortical T2 values (**D**); medullary T2 values (**E**); and T2 CMD (**F**)) and *e*GFR. The third row (**G**–**I**) shows the correlation between ADC parameters (cortical mean ADC values (**G**); medullary mean ADC values (**H**); and ADC CMD (**I**)) and eGFR. The fourth row (**J**–**L**) shows the correlation between FA parameters (cortical mean FA values (**J**); medullary mean FA values (**K**); and FA CMD (**L**)) and *e*GFR. The best correlations with *e*GFR could be obtained for renal cortical T1 values (*r* = −0.63, **A**), for mean medullary FA values and *e*GRF (*r* = 0.67 (**K**)) and for FA CMD (*r* = 0.62 (**L**)). In comparison to T1 and FA values, T2 and medullary ADC values showed significant, but low to negligible correlations (*r* between 0.14 and 0.35). Blue colors of the points indicate higher *e*GFR.

Cortical T1 relaxation times negatively correlated with *e*GFR on the day of the MRI examination, whereas the CMD showed a positive correlation with *e*GFR. While the strength of correlation between cortical T1 and *e*GFR was moderate (*r* = −0.63), it was weaker for T1 CMD, and *e*GFR (*r* = 0.47). Regarding T2 parameters, all parameters only showed weak to negligible correlations with renal function, whereby the highest correlation was between medullary T2 and *e*GRF with a correlation coefficient of −0.35. The best correlation between eGRF and FA parameters was found for mean medullary FA values (*r* = 0.67, *p* < 0.001) as well as FA CMD (*r* = 0.62, *p* < 0.001). Correlations between eGFR and cortical FA as well as ADC parameters were significant, but weak (*r* between −0.14 and 0.35). Figure 3 shows an overview of the correlations of T1, T2, ADC, and FA values with *e*GFR.

### 3.5. Diagnostic Performance of MRR and DTI Parameters Compared to Laboratory Markers of Renal Function and Biopsy Results

**Figure 4:**
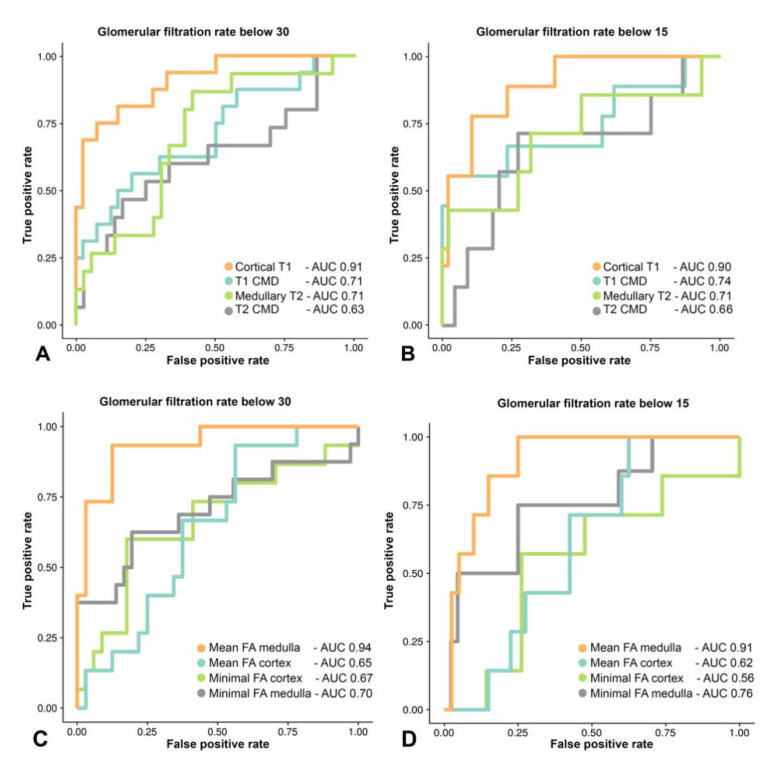
The Area under the Receiver Operating Characteristic Curve (AUC) for distinguishing glomerular filtration rates below 30 (**A**,**C**) and below 15 (**B**,**D**) from better functioning kidneys was highest for cortical T1 (AUC of 0.91 or 0.90) (**A**,**B**) and mean medullary FA values (AUC of 0.94 or 0.91) (**C**,**D**).

In a receiver operating characteristic curve (ROC) analysis to establish the suitability of different MRR and DTI parameters for the assessment of high (*e*GFR below 30 mL/min) and severe renal insufficiency (*e*GFR below 15 mL/min), we found that the area under the ROC (AUC) was highest for cortical T1 (AUC of 0.91 or 0.90) and medullary FA (AUC of 0.94 or 0.91) (cf. Figure 4). Cortical T1 and medullary FA therefore appear to be the best predictors/classifiers of terminal renal failure as defined by *e*GFR below 30 mL/min or 15 mL/min.

By comparison, for T1 CMD the AUCs were 0.71 to distinguish an eGRF below 30 and 0.74 to differentiate an eGRF below 15 from better functioning kidneys. For medullary T2, the AUCs were 0.71 to distinguish an eGRF below 30 or below 15 from better functioning kidneys. Cortical FA reached an AUC of 0.67.

Seven biopsies were available for analysis (all of them dysfunctional allografts; 6/7 with tubular atrophy and 7/7 with interstitial fibrosis). In a logistic regression analysis, mean medullary FA proved to be a significant predictor for tubular atrophy (*p* < 0.001), while cortical T1 appeared as a significant predictor of interstitial fibrosis (*p* = 0.003).

### 3.6. Longitudinal Subgroup

Results from the small subgroup of six early transplants who were measured twice can be accessed in Table 3. T1 values generally show a decrease between the first and second measurement, while there are no apparent differences for T2 values. ADC values and medullary FA values demonstrate an increase between the first and second measurement. Differences between mean cortical FA values are less pronounced.

## 4. Discussion

After kidney transplantation, T1 and T2 relaxometry showed an increase of T1 and T2 relaxation times and a loss of CMD in patients compared to healthy controls. Conversely, cortical and medullary FA values as well as ADC values were decreased in early transplants. In general, renal changes were more pronounced in early transplants compared to late transplants. However, even in the longer term after surgery, kidney transplants showed tissue-specific values that differed from those of healthy, non-transplant controls. This study suggests that quantitative cortical T1 and medullary FA show the best correlation with eGFR, as well as with histopathology (interstitial fibrosis and tubular atrophy) in a small number of available biopsies. Hence, multiparametric MRI might enable the non-invasive assessment of renal tissue changes following renal transplantation.

To date, the inconsistency of imaging protocols remains an ongoing challenge for multiparametric renal MRI, which requires standardization. Recently, recommendation papers have been published for both renal relaxometry and DWI [20,21]. The resulting recommendations for the clinical translation of T1 and T2 mapping based on the opinions of 18 experts include normal hydration, measurements at field strengths of 1.5 or 3 T and a coronal or coronal oblique orientation [20]. For T1 mapping, MOLLI is the recommended acquisition scheme with more than one slice, at least 3 mm in-plane resolution, flip angle of 35°, parallel imaging factor of 2, and breath-holds of less than 15 s [20]. Furthermore, the collection of data with fixed spacing instead of ECG gating is favored (85% agreement) [20]. For T2 mapping, a minimum of five echo times is suggested for data collection as well as maximum echo times and high T2 preparation times (e.g., 120 ms) [20]. In addition, Dekkers et al. summarized recommendations regarding the analysis and reporting of T1 and T2 values [20]. The protocol of this study is consistent with the recommendation in all but two aspects (one discrepancy for T1 and T2 mapping each). First, ECG gating instead of fixed spacing was used for T1 mapping. Although from our experience, ECG gating with a trigger delay adjusted to the diastolic period of cardiac motion provided good results for kidney transplants, which are generally less affected by respiratory movement due to their location in the iliac fossa, it might have been inferior to fixed spacing in healthy controls. In the general interest of protocol standardization, it would also have been advantageous to use fixed spacing in order to standardize the protocols. Second, even though the recommended five preparation times were used, they were lower than the recommended 120 ms. Due to the relatively long T2 relaxation times of the kidneys, ideally longer T2 preparation times are used. Hence, the T2 maps in the present study are partly influenced by renal T1 signal and do not correspond to a pure T2 signal. This is also discussed in the limitations section.

Recent recommendations for the clinical translation of renal diffusion-weighted MRI comprise normal hydration of the patients, single shot EPI, oblique coronal orientation, matrix >128, in-plane resolution of 2–3 mm, full kidney coverage, spectral attenuated inversion recovery fat saturation, TE <100, distortion correction, unilateral registration, if possible, and metric statistics reporting with mean, median, standard deviation, and ROI size [21]. Regarding the choice of b-values and diffusion directions, consensus was found for more than two b values, including values <200 mm/s^2^, and for more than 12 diffusion directions [21]. Manual ROI placement had consensus support over automatic placement [21]. Again, the protocol of the present study is consistent with these recommendations in all but two aspects: First, DTI was not acquired in oblique coronal, but in axial orientation. However, since the sequence was acquired with an isotropic voxel size (2.7 mm), it could be reformatted in any desired plane. Second, DTI was only acquired with two b values of 0 and 600 mm/s^2^. While a minimum of two b values is enough to reach a stable diffusion signal, including more b values in the acquisition protocol allows for a more precise description of diffusion signal decay and could improve the robustness of DTI [21,22]. Therefore, the choice of only two b values in the present study has to be pointed out as a potential limitation. Another important aspect of DTI the acquisition of multiple diffusion directions for tensor computation, given that diffusion anisotropy is the key imaging target [21]. As we chose a total of multiple diffusion directions in our protocol (*n* = 64), it can be assumed that this lowered the variability for the estimation of FA [23].

In the context of T1 and T2 relaxometry, animal research suggested an association of T2 relaxation times with renal edema after interruption of blood flow and that increased water content is an important cause for the prolongation in renal T1 relaxation times [24,25]. Friedli et al. applied histological validation of quantitative T1 relaxometry in mice and patients with renal transplants, finding that state-of-the-art T1 measurements, such as T1 CMD, could be used for assessment of renal interstitial fibrosis in allografts [17]. The reason for a reduction of T1 CMD in case of graft dysfunction results from an increase of T1 relaxation times in the cortex, while the medulla only shows a mild increase. It was previously suggested that the less pronounced increase in the renal medulla is a result of the reduced water content in the tubular structures due to renal impairment [26]. Similar to the present study, Friedli et al. reported a correlation between T1 values and interstitial fibrosis. In clinical research, Huang et al. found higher cortical T1 values in transplanted kidneys compared to native kidneys [27], but they only examined a small number of patients with varying time since transplantation. Peperhove et al. investigated patients within two weeks after kidney transplantation with T1 relaxometry at a field strength of 1.5 T, reporting an increase in renal T1 compared to healthy volunteers as well as a reduction of CMD shortly after transplantation, yet they did not examine renal T2 and only evaluated early transplants [26]. Bane et al. investigated 27 renal transplants with stable and instable function and suggested, that a combination of cortical ADC and T1 measurements showed promise for the non-invasive assessment of renal allograft histology and outcomes [28]. As an extension to this aforementioned work, the present study additionally examines T2 relaxometry and does not only investigate early transplants, but also late transplants. In addition, there is a small sub-study on longitudinal postoperative changes. Finally, the current study uses a field strength of 3 T instead of 1.5 T, which may provide higher sensitivity to changes in T1 for detecting renal tissue changes.

For DWI, Wang et al. found that functional ADC measurements correlated with kidney allograft interstitial fibrosis. In the context of DTI, studies in human kidneys demonstrated higher FA values in the renal medulla compared to the renal cortex [29,30]. A recent study on renal transplants suggested a decreased mean FA of the renal cortex and medulla in allografts with impaired function compared to better functioning allografts [31]. The observed changes in ADC and FA with decreased ADC and especially FA values in early and dysfunctional transplants may result from underlying pathological changes, such as cell swelling and capillary leakage in early transplants or renal fibrosis with interstitial collagen deposition in dysfunctional transplants. Both contribute to a restriction of tissue diffusivity, decreasing the Brownian motion of water molecules by collision [15]. Renal tubules, collecting ducts, and vessels are very orientated structures, giving strong diffusion preference radial to the renal pelvis [32]. Hence, pathological changes could influence directed diffusion (FA) before global diffusion (ADC) is affected, which could serve as a possible explanation for the better association of medullary FA with eGFR and histopathology when compared to ADC. The FA values reported in the present study for kidney transplants are similar to the ones reported in prior research [33]. In contrast to previous studies, the present study used 64 diffusion directions (compared to a minimum of 12), providing an optimal quantification of FA. The quantitative T1 values we obtained were comparable to those reported in the literature for late transplants, even though it has to be noted that there was only one previous study investigating renal transplants with a field strength of 3 T [17]. With regard to early transplants, the current study is the first to report the corresponding T1 values using a field strength of 3 T.

Even though we found significant correlations between T2 and ADC parameters and eGFR, these correlations were comparatively weak. In addition, for these parameters, we did not observe any significant correlations with histopathology. Therefore, based on our results, T2 relaxometry appears inferior to T1 relaxometry for the evaluation of renal function as measured by eGFR, while—in agreement with Bane et al.—DWI seemed inferior to DTI. However, it has to be pointed out that no recommendations for the most suitable relaxometry or diffusion parameters to predicted outcomes of biopsies or to identify high- or low-risk groups can be derived from the results of the present study. Obviously, the strong correlation with eGFR observed for T1- and FA-derived parameters does not allow for an estimate of renal function superior to eGFR. The number of available biopsies, all taken from malfunctioning kidney, was very low (*n* = 7). Future studies on multiparametric MRI should therefore focus on the correlation of quantitative MR parameters with histopathological results as an indicator for clinical outcomes. As a first step towards larger diagnostic and prognostic studies, the repeatability of multiparametric kidney MRI has to be determined [34]. Intersession repeatability for multiparametric renal imaging was previously investigated, with a coefficients of variation (CoV) of 2.8–5.1% for T1 mapping, 2.9% for T2 mapping, and 2.8–6.7% for DTI [35]. However, for our scanner and imaging protocol, intersession reproducibility was not assessed, which is an important limitation.

Apart from this, the present study has some further limitations: First, there is no long-term follow up, which prevents insight into the detailed prognostic value of T1/T2 relaxometry and DTI. Another aspect is that MOLLI was performed with cardiac triggering. Although MOLLI with cardiac triggering is frequently used for cardiac imaging, the recent consensus-based recommendations by Dekkers et al. instead proposed a fixed interval of 1 ms between measurements for renal imaging [20]. Besides, only two b values were used for DTI. Furthermore, the control group was slightly younger than the group of subjects with renal transplants, and thus not perfectly age-matched. In addition, the group of patients with long-term kidney transplants was heterogeneous, ranging from 6 months to 13 years. Finally, the number of biopsies was very limited (*n* = 7) and the time intervals between biopsies and MRI varied. In addition, the longest time interval between biopsy and MRI was nearly six months, which could make the comparison to MRI unreliable. To this end, the observed correlations between histology and MRI must be viewed critically. Future studies should therefore evaluate to what extent MRR parameters could complement the laboratory values and if they correlate with histology.

## 5. Conclusions

In summary, quantitative T1 and T2 relaxation times are most increased, while ADC and FA values are most decreased in early transplants. They appear to remain, respectively, higher (T1 and T1) or lower (ADC and FA) in the long term compared to healthy subjects, suggesting permanent underlying tissue changes. Out of all functional parameters, cortical T1 and medullary FA showed the best correlation with eGFR. In a small number of available biopsies, they were also associated with interstitial fibrosis (T1) and tubular atrophy (FA). Consequently, cortical T1 and medullary FA might serve as new imaging biomarkers of renal function and histopathologic microstructure.

## Figures and Tables

**Table 1:** Tabulated MR imaging parameters.

Sequence	T2 Single-Shot FSE *	Dixon	T1 Mapping	T2 Mapping	DTI
Scan plane	Oblique coronal	Oblique coronal	Oblique coronal	Oblique coronal	Axial
Voxel size (mm)	0.7 × 0.7 × 4.0	1.6 × 1.6 × 1.3	0.8 × 0.8 × 4.0	1.9 × 1.9 × 4.0	2.7 × 2.7 × 2.7
Acquisition time (min)	1:36	00:23	3:33	1:51	8:14
Number of slices	31	31	19	7	50
TR/TE (ms)	1200/94	4.21/1.26; 2.49	551.28/1.35	766.29/1.44	6400/75
Averages	1	1	1	1	
FoV (mm)	350	400	350	360	350
Flip angle (°)	153	9	35	12	90
Matrix	512	256	224	192	128
Bandwidth (Hz/Px)	407	780	1063	1184	1698
Fat saturation	None/Yes	None	None	None	Strong
Number of preparations (duration in ms)			2	5 (0, 30, 34, 38, 42)	
Trigger delay (ms)			379	164	
Breath-holding procedures			19	7	12
b-values					2 (0, 600)
Diffusion directions					64 (2 signals acquired)
Echo spacing (ms)	5.18		3.04	3.29	0.65

* FSE, fast spin echo.

**Table 2:** Overview of study characteristics.

	Early Transplants	Late Transplants	Controls
Number (men/women)	22 (17/5)	24 (20/4)	16 (10/6)
Age (years) ± SD	54.90 ± 0.13	51.26 ± 12.94	59.9 ± 14.4
eGFR (mL/min) ± SD	31.54 ± 4.95	47.48 ± 12.73	80.53 ± 21.43
Time since transplantation	6.8 ± 3.4 days	5.5 ± 5.1 years	NA
T1 in ms ± SD (cortex, medulla)	1700 ± 53, 2048 ± 72	1615 ± 47, 2004 ± 68	1514 ± 29, 1939 ± 51
T2 in ms ± SD (cortex, medulla)	83 ± 6, 67 ± 5	82 ± 8, 62 ± 3	78 ± 4, 59 ± 2
Mean FA ± SD (cortex, medulla)	0.085 ± 0.006, 0.310 ± 0.006	0.093 ± 0.008, 0.318 ± 0.022	0.108 ± 0.005, 0.401 ± 0.006
ADC in 10^−6^ mm^2^/s ± SD (cortex, medulla)	2130 ± 45, 1834 ± 40	2189 ± 54, 1988 ± 63	2269 ± 48, 2051 ± 49

**Table 3:** Data on the longitudinal subgroup.

Patient	T1 Map Cortex (ms)		T1 Map Medulla (ms)		T2 Map Cortex (ms)		T2 Map Medulla (ms)	
	TP 1	TP 2	TP 1	TP 2	TP 1	TP 2	TP 1	TP 2
1	1710	1617	1872	1884	88	79	63	56
2	1582	1584	1963	1924	91	96	79	83
3	2005	1707	2224	2030	80	95	72	75
4	1736	1638	2135	2057	81	88	64	67
5	1882	1691	2088	1975	77	70	66	59
6	1675	1413	1974	1839	70	85	63	51
**Patient**	**Mean ADC Cortex** **(10^−6^ mm^2^/s)**		**Mean ADC Medulla** **(10^−6^ mm^2^/s)**		**Mean FA Cortex**		**Mean FA Medulla**	
	**TP 1**	**TP 2**	**TP 1**	**TP 2**	**TP 1**	**TP 2**	**TP 1**	**TP 2**
1	1760	2222	1556	2029	0.161	0.152	0.210	0.333
2	2250	2342	1941	2207	0.057	0.106	0.279	0.336
3	2137	2411	1650	1843	0.117	0.068	0.209	0.307
4	2293	2329	1565	1983	0.072	0.079	0.310	0.381
5	2028	2296	1860	2100	0.092	0.136	0.213	0.226
6	2141	2337	2327	2018	0.053	0.072	0.204	0.403

TP, time point. The six patients of the longitudinal group were examined at two time. points shortly after the transplantation (TP 1 and TP 2).

## Supplementary Materials

The following are available online at https://www.mdpi.com/2077-0383/9/5/1551/s1, Figure S1: Regions of interest (ROI) placement.
