# Supplementary material for: Multiparametric Assessment of Changes in Renal Tissue after Kidney Transplantation with Quantitative MR Relaxometry and Diffusion-Tensor Imaging at 3 T
Source: J Clin Med. 2020 May 21;9(5):1551. doi: 10.3390/jcm9051551 (PMC7290480; doi:10.3390/jcm9051551)
Supplement: Supplementary file 1 [file jcm-09-01551-s001.pdf]

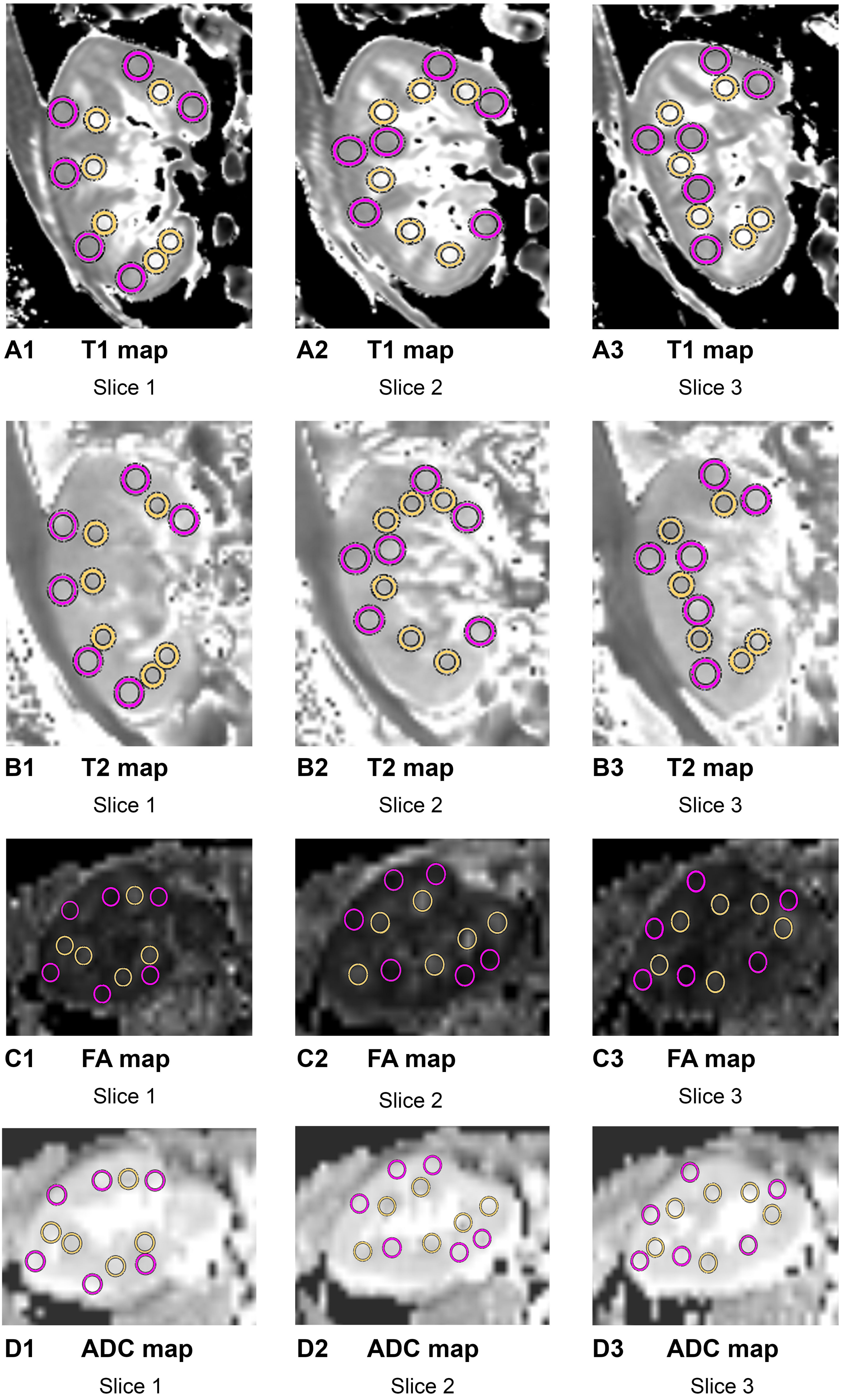

A1 T1 map
Slice 1
A2 T1 map
Slice 2
A3 T1 map
Slice 3
B1 T2 map
Slice 1
B2 T2 map
Slice 2
B3 T2 map
Slice 3
C1 FA map
Slice 1
C2 FA map
Slice 2
C3 FA map
Slice 3
D1 ADC map
Slice 1
D2 ADC map
Slice 2
D3 ADC map
Slice 3
